# Dispersants as Used in Response to the MC252-Spill Lead to Higher Mobility of Polycyclic Aromatic Hydrocarbons in Oil-Contaminated Gulf of Mexico Sand

**DOI:** 10.1371/journal.pone.0050549

**Published:** 2012-11-27

**Authors:** Alissa Zuijdgeest, Markus Huettel

**Affiliations:** 1 Department of Earth Sciences, Utrecht University, Utrecht, The Netherlands; 2 Department of Earth, Ocean and Atmospheric Science, Florida State University, Tallahassee, Florida, United States of America; University of California, Merced, United States of America

## Abstract

After the explosion of the Deepwater Horizon oil rig, large volumes of crude oil were washed onto and embedded in the sandy beaches and sublittoral sands of the Northern Gulf of Mexico. Some of this oil was mechanically or chemically dispersed before reaching the shore. With a set of laboratory-column experiments we show that the addition of chemical dispersants (Corexit 9500A) increases the mobility of polycyclic aromatic hydrocarbons (PAHs) in saturated permeable sediments by up to two orders of magnitude. Distribution and concentrations of PAHs, measured in the solid phase and effluent water of the columns using GC/MS, revealed that the mobility of the PAHs depended on their hydrophobicity and was species specific also in the presence of dispersant. Deepest penetration was observed for acenaphthylene and phenanthrene. Flushing of the columns with seawater after percolation of the oiled water resulted in enhanced movement by remobilization of retained PAHs. An in-situ benthic chamber experiment demonstrated that aromatic hydrocarbons are transported into permeable sublittoral sediment, emphasizing the relevance of our laboratory column experiments in natural settings. We conclude that the addition of dispersants permits crude oil components to penetrate faster and deeper into permeable saturated sands, where anaerobic conditions may slow degradation of these compounds, thus extending the persistence of potentially harmful PAHs in the marine environment. Application of dispersants in nearshore oil spills should take into account enhanced penetration depths into saturated sands as this may entail potential threats to the groundwater.

## Introduction

Roughly 780000 m^3^ light crude oil were released into the Gulf of Mexico after the blowout at the drilling rig Deepwater Horizon (MC252 well) in April 2010 [1], [2]. In order to disperse the oil, to prevent a surface slick from accumulating, and to aid biodegradation [3], dispersants were applied both at the surface (6800 m^3^, Corexit 9500A and Corexit EC9527) and in the subsurface close to the wellhead (3000 m^3^, Corexit 9500A only) [4]. After initiation of the surface applications, dispersants were present in coastal waters of the northeastern Gulf of Mexico at least until 18^th^ October 2010, and the OSAT 1 [4] report lists 60 nearshore water samples and 6 nearshore sediment samples in which dispersant was found.

Dispersants cause disintegration of oil accumulations, creating smaller droplets with high surface-to-volume ratios, which are more accessible and easier to degrade by microorganisms [3], [5], [6]. Hazen et al. [7] found increased numbers of hydrocarbon-degrading microorganisms in Gulf water containing dispersed oil, and Kostka et al. [8] reported fast and extensive changes in the bacterial composition in oil-contaminated Florida beaches towards more hydrocarbon- degrading species. However, in several other studies, dispersed oil was not degraded faster than undispersed oil [9], [10].

Relatively little is known about the environmental effects and the fate of dispersants applied to marine oil spills [11]. According to Wise and Wise [12], 38 peer-reviewed articles were available on the toxicity of 35 different chemical dispersants as of February 2011, with several studies indicating toxicity effects to marine organisms. Grattan et al. [13] found increased mortality in planktonic copepods exposed to dispersants with stronger effects on small-sized species. In a study with early life stages of Atlantic herring, dispersed oil dramatically impaired fertilization success [14]. Flin [15] reported that grey mullet exposed to chemically dispersed oil showed both a higher bioconcentration of polycyclic aromatic hydrocarbons (PAHs) and a higher mortality than fish exposed to either the water-soluble fraction of oil or the mechanically dispersed oil. No dispersants were used in the cleanup of the Exxon Valdez spill [16], which could have provided some insights in dispersant effects on oil-contaminated beaches. In the Gulf of Mexico, the application of Corexit™ was successful in terms of dispersing a fraction of the oil, some of which formed subsurface plumes [17], [18]. Recent data [19] shows that dispersant applied at the wellhead did not surface, and that the concentrations measured in the upper water column were due to the massive surface applications. The subsurface Corexit application contributed to lower oil concentrations in the surface layer than originally expected [16], [20], nonetheless, substantial volumes of oil reached the coast of the northeastern Gulf of Mexico [4].

MC252 oil that reached the shallow nearshore zone became subject to enhanced dispersion by breaking waves that disintegrated and mixed oil parcels throughout the shallow-water column. Bottom currents can rapidly transport dissolved and particulate matter into permeable sublittoral sands [21], [22], thus providing a pathway for oil components into the seabed. Water that washed up the beach face by waves carried dispersed oil onto permeable saturated sands. When a wave recedes from a beach face, the ensuing water level drop within the highly permeable beach sand draws large volumes of water into the beach face [23], [24]. The question arises whether this water penetration into the permeable sands could transport dispersed oil components into the sand and if so, how deep. So far no data were available showing whether the application of Corexit affected the potential of oil components to enter and migrate through the pore space of permeable saturated Gulf of Mexico sands. Dispersants reduce oil-droplet size and thereby increase mobility of hydrophobic compounds in the water phase through the formation of small micelles [25], [26]. These micelles can be as small as a few micrometers in diameter [27], which may facilitate deep penetration of dispersed-oil hydrocarbons into beach sands with pore water flows.

The possible transport of oil components into the beach presents an environmental risk factor because crude oil components, for example several PAHs, (e.g. benzo(a)anthracene, chrysene, benzo(b)fluoranthene, benzo(a)pyrene and benzo(ghi)perylene), are known to be potential human carcinogens [28] and to affect marine larvae [29], [30] and filter feeders that can bioaccumulate PAHs [31]. In water samples collected in the Gulf of Mexico in June 2010, after the Deepwater Horizon Accident, Allan et al. [32] found PAH concentrations up to 170 ng L^−1^, which was significantly greater than the baseline concentrations observed before the accident. Oil from the Prestige accident resulted in Spanish beach sediment PAH concentrations of 0.9–422 µg kg^−1^ dw [33] that affected sedimentary organisms [34]. PAHs have a high affinity for sediments, and are characterized by low volatility and resistance to decomposition [35]. Due to their slow degradation rates, some of these PAHs are persistent components after oil spills [36], e.g. White et al. [37] reported that PAHs originating from the 1969 oil spill in Buzzards Bay, MA, [38]–[40] still persist in the sediments at concentrations of up to 134 mg kg^−1^. Sediment organic matter and weathered oil content amplify adsorption of PAH in sediments [41], [42], and thereby decrease PAH transport and degradation rates [43], [44]. PAH degradation can proceed aerobically [45], but not under strictly anaerobic conditions [46]–[51], though decomposition was observed at reduced rates in presence of nitrate or sulfate [52]–[55]. Half-lives determined for PAHs in soils range from days to decades [56].

A wealth of information on the effect of surfactants on the fate of PAHs in aqueous and sedimentary environments is presented in research addressing PAH mobilization and decomposition in soils and laboratory cultures. In the latter, surfactant enhanced the bioavailability of PAH mixtures to *Pseudomonas putida* [57] and *Stenotrophomonas maltophilia* [58], and such surfactant-enhanced PAH degradation was further confirmed by [59], [60]. Recent work has shown that a fraction of PAH solubilized by surfactants can be transferred directly from the core of the surfactant micelle to the microbial cell without having to transfer to the water phase first, thereby accelerating PAH decomposition [61]. In contrast, some studies also have shown that surfactant addition can inhibit PAH biodegradation via toxic interactions or sequestration of PAHs into surfactant micelles [62]. Tiehm [63] found that the degradation of PAHs was inhibited by sodium dodecyl sulfate because this surfactant was preferred as a growth substrate. Studies in soils show that surfactants affect the distribution of hydrophobic organic compounds in the soil/aqueous system [64]–[66]. Surfactants solubilize the PAHs in soils, and this process is proportional to the surfactant concentration [67], [68]. PAH solubilization takes place at the sediment/oil-water interface and also within the oil-saturated soil matrix because surfactants penetrate into the matrix thereby increasing diffusivity of PAHs [69].

Application of dispersants after the Deepwater Horizon accident, thus, may have also increased the intrusion and penetration of PAHs into Gulf of Mexico saturated sands, thereby creating a potential threat to groundwater through PAH contamination. Despite this potential threat, data showing whether and how PAHs can enter and travel through wave-flushed saturated Gulf sands affected by dispersant application are not available, precluding an assessment of the environmental risks associated with this application. The goal of this study therefore was to quantify intrusion and migration of PAHs contained in dispersed oil with and without Corexit addition in permeable water-saturated Florida sands sediments. To this end, we used laboratory column reactors containing natural saturated beach sand that were percolated by defined volumes of seawater with known concentrations of MC252 oil and Corexit 9500A, and measured distribution and concentrations of the dominant PAHs, in the pore water and effluent of the columns using GC/MS. Two benthic chamber experiments, designed to link laboratory results to natural settings, tested whether oil components are transported into permeable sublittoral Gulf sands. Our working hypothesis was that Corexit would increase the mobility of the PAHs in the saturated sand, thereby increasing their flux, penetration depth and dispersion throughout the sand. The results of the study show that the presence of dispersant significantly increases the mobility and penetrations depth of PAHs in saturated Gulf beach sands.

## Methods

### Study Site and Permit

Sediment collections and chamber experiments took place at Santa Rosa Island near Pensacola Beach, Florida. After the Deepwater Horizon accident, oil was deposited on the sandy beaches of this island and some of the oil was buried in the beach sands. A Scientific Research and Collection Permit (GUIS-20100SCI-0025) for this study was obtained from the United States Department of the Interior, National Park Service, Gulf Islands NS. The research took place at public lands and no protected species were sampled.

### Short-column Experiments

**Figure 1:**
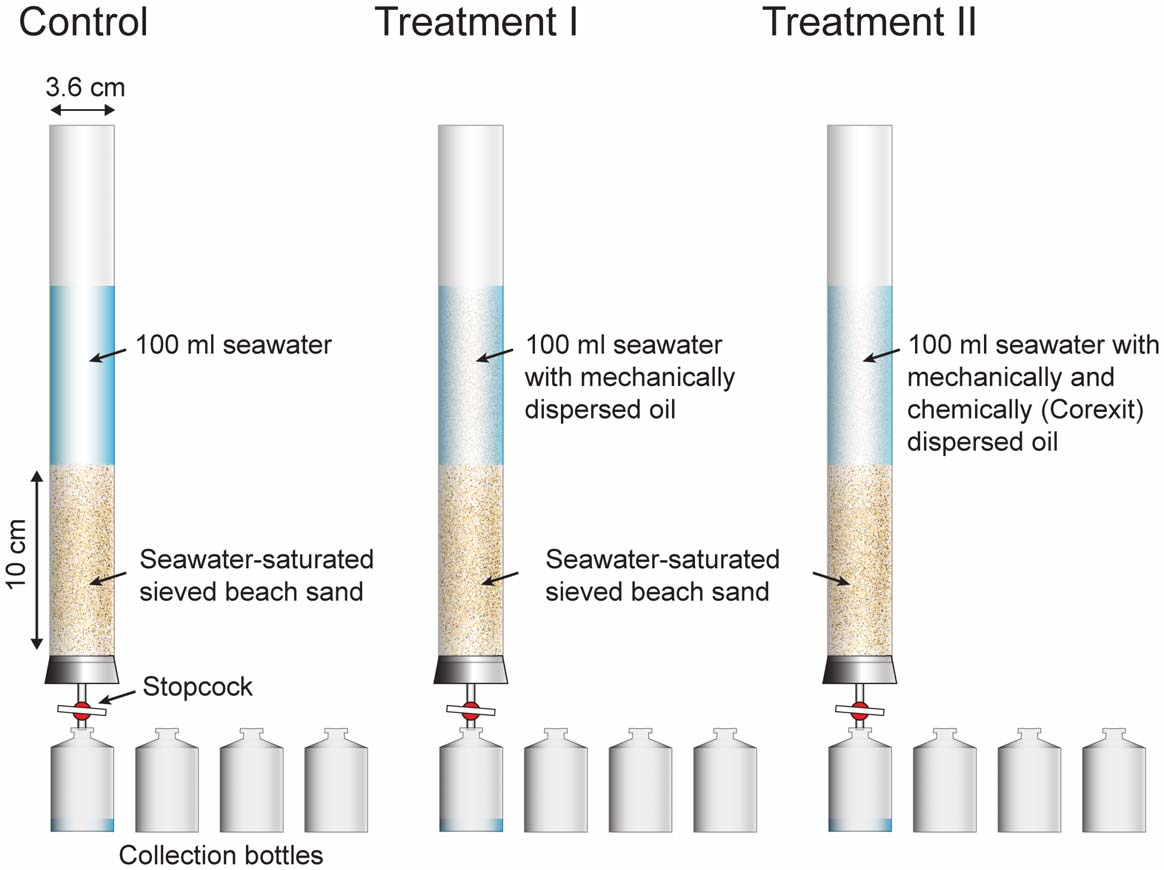
Setup of the Short-Column Experiment. Clean seawater, crude oil dispersed by sonication, or crude oil dispersed by Corexit and sonication were flushed through the sand columns by gravity. The effluent of the columns was collected as a time series in 4 vials each.

The laboratory column reactors used for this first experiment consisted of transparent acrylic core liners (3.6 cm i.diam., 30 cm long) that were sealed at the bottom with gauze-covered stoppers preventing sand from clogging the stopcock that controlled the outflow of the columns (Fig. 1). Oil-free sand, collected following recommended methods [70] from the upper part of the public beach at Pensacola Beach, Florida, (30°19′33.96′′N, 87°10′28.87′′W), was sieved to remove shell hash and large organic debris. The sand fraction smaller 500 µm was filled into the core liners to produce 10 cm sand columns saturated with artificial seawater (S = 33, created with Instant Ocean®). This procedure resulted in sand columns with similar permeabilities allowing comparison of the columns and quantifiable percolation rates. Three series of experiments were conducted that differed in their composition of the percolating water (Table 1).

**Table 1:** Composition of the percolating water in the Short- and Long-Column Experiments.

Control	100 mL artificial seawater (+ rinse with 300 ml artificial seawater)
Treatment I	100 mL artificial seawater with 1 mL oil dispersed into small droplets by ultrasound treatment (+ rinse with 300 ml artificial seawater).
Treatment II	100 mL artificial seawater with 1 mL oil dispersed by Corexit 9500A addition at 1∶100 dispersant to oil ratio and ultrasound treatment (+ rinse with 300 ml artificial seawater).

The long columns were subsequently flushed with an additional 300 mL of clean artificial seawater (in parenthesis).

The crude oil used for these experiments was MC252 oil that had been collected from the leaking wellhead after the Deepwater Horizon explosion and was made available to the scientific community by the Gulf of Mexico Research Initiative. Corexit 9500A is produced by NALCO and commercially available. All experiments were conducted in duplicate. After removing all seawater above the saturated sand contained in the column reactors, 100 ml of the fluids listed in Table 1 were added slowly (to not resuspend sand) to the columns. Immediately after this addition was completed, the stopcock at the exit of each column was opened, allowing the water overlying the sand columns to percolate through the sands by gravitation, similar to the process occurring when water washed up the beach is penetrating into the beach face by gravity. All water exiting each column was collected as 4 separate samples, each between 20 and 30 mL, to obtain a time series of effluent PAH concentrations. The experiment was stopped for each column when the water level overlying the sand column reached approximately the respective sediment surface. After the fluorescence measurements, the sand columns were sectioned at 2.5 cm intervals and the hydrocarbons were extracted from each layer using standard procedures explained below. In order to assess the effect of Corexit on PAHs contained in oil emulsified in seawater, 5 ml oil dispersed either mechanically (ultrasound treatment for 2 minutes) or mechanically and chemically (addition of 50 µL Corexit, ultrasound treatment for 2 minutes), were thoroughly mixed with 8 L of natural seawater each, and after 24 h dark incubation in the dark at 22°C, samples were extracted for PAH analysis.

### Long-sand Column Experiments

In the second experiment, we used sand columns of 10, 25 and 50 cm lengths in order to assess the distance PAHs can travel through saturated Gulf of Mexico sand. The experiment again was initiated by opening the stopcocks at the exit of the columns allowing the water overlying the sands to percolate the sand columns by gravitation. The fluids that were flushed through these columns initially were of same volume and composition as for the first experiment (Table 1). When the dropping water level in the columns reached the sediment surface, flow was stopped and an additional 300 mL of clean artificial seawater was added to each column. This ensured that the pore space of all columns (approximately 1/3 of the sand volume) was flushed completely. All water of each column was collected in a 500 mL flask, and care was taken to minimize evaporation and contact to the atmosphere.

### Analytical Procedures

#### Fluorescence measurements

In order to get a rough estimate of the depth of oil penetration through the sand columns, the distribution of fluorescence in the sand was measured through the transparent core liners. The aromatic hydrocarbons contained in oil fluoresce [71] and, thus, can be measured as chromophoric dissolved organic matter (CDOM) [72], [73]. Immediately after closing the column outflows, a fiber-optic probe emitting pulses of blue-enriched light (λ<710 nm) was used to measure fluorescence at different sediment depths. Fluorescence produced by oil-stained sand was returned through the fiber-optic cable to a photodiode protected by a long-pass filter (λ<710 nm). The amplified output signal of the photodiode produced the measuring signal. In order to account for uneven penetration of oil through the sand, each sand column was scanned along its entire length three times at 0.5 cm measuring intervals on four sides of the column. Each scanning of an experimental core was followed by a scan of a calibration core used to convert the averaged fluorescence measurements for each depth interval into oil concentrations. This calibration core was prepared with a core liner of the same diameter and the same sand as used in the column experiment, and contained a series of 5 cm thick sand sections differing in known oil concentration, covering the range between 0 and 70 mg oil g^−1^ dry sand.

#### Water samples

Hydrocarbons were extracted from water samples using a liquid-liquid extraction according EPA method 3510 adapted for small sample volumes. Extraction was done with 3×10 mL dichloromethane (DCM) in a 125 mL separatory funnel, then samples were subjected to the EPA 3630C silica cleanup method. A glass column with an internal diameter of 1.1 cm was packed with 10 centimeters of activated (2 h at 160°C) silica, topped with 1 cm sodium sulfate. The column was saturated with DCM and subsequently pre-eluted with 40 mL pentane. Afterwards, 2 ml sample was transferred to the column using an additional 2 mL pentane to complete the transfer. The sample then was flushed through the column with a subsequent addition of 25 mL pentane to move all sample into the column. Collection of the analyte was initiated by changing the eluting solvent to a DCM:pentane mixture (2∶3 v/v, 25 mL). The collected sample was concentrated to a final volume of ∼2 mL and filled in capped vials for the Gas Chromatograph/Flame Ionization Detector (GC/FID) and Gas Chromatograph Mass Spectrometer (GC/MS) analyses described below. The water samples for dissolved inorganic carbon (DIC) were filtered through GF-F filters to minimize particulate hydrocarbon influence and analyzed in duplicate on a Shimadzu TOC-VCPH.

#### Sediment samples

**Figure 2:**
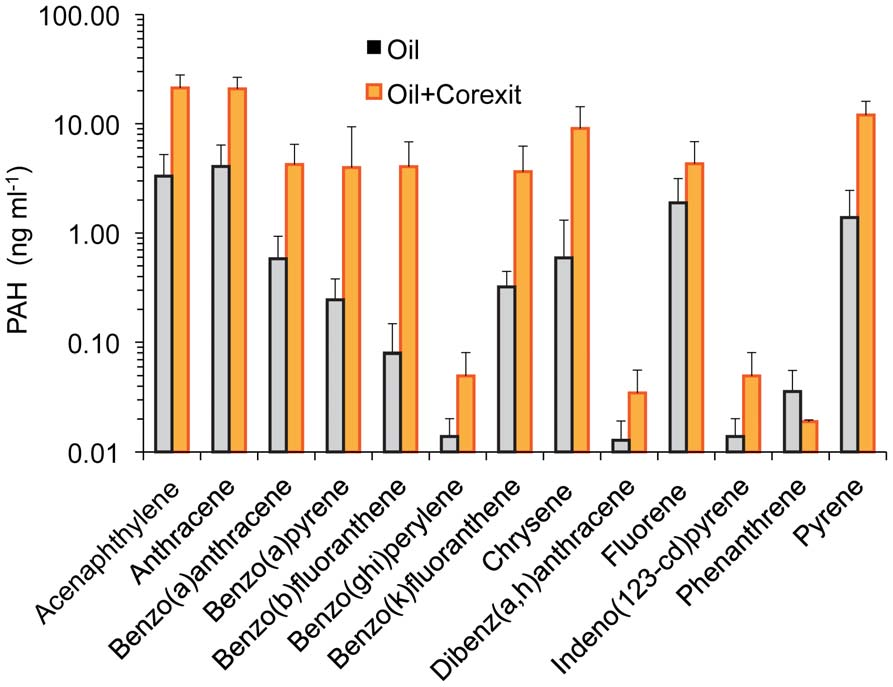
PAH concentrations in seawater after the addition of oil or oil and Corexit. PAH concentrations were measured in the seawater 24 h after the addition of Deepwater Horizon Crude oil or addition of the same amount of oil with Corexit. Note logarithmic Y-axis scaling.

After the experiments, the sand columns were sectioned at 2.5 cm depth intervals, and each depth section was thoroughly mixed. Hydrocarbons were extracted from a 10 g subsample of each homogenized section using an automated Behr-E4 Soxhlet extraction system according to EPA method 3541. The one-hour extraction with a hexane:acetone 1∶1 v/v mixture was followed by one hour rinsing with the same solvent mixture. The solvents containing the extracts were concentrated using a Turbovap-II rapid solvent evaporator (55°C) to less than 5 mL, and then transferred to cyclohexane by adding 20 mL of that solvent. Concentration was continued until the sample was reduced to roughly a volume of 2 ml that was subjected to the same clean up procedures as explained for the hydrocarbons extracted from the water samples. For the calculation of recovery rates and background values, several blanks and spiked samples were analyzed using the same protocol as for the other samples. The recovery rates for the PAHs were 86 to 112% for the water extraction and 65 to 110% for the sediment extraction, and were within the limits of the EPA protocol. Average water PAH background value for the 12 PAHs that were quantified in the water used for the column experiments was 0.02±0.04 (SD) ng l^−1^ (short column experiment) and 0.25±0.32 (SD) ng l^−1^ (long column experiment). PAH background values for the sediment were 2.60±7.74 (SD) ng kg^−1^ (short column experiment) and 3.23±3.71 (SD) ng l^−1^ (long column experiment). The variability of PAH concentrations between parallel samples is shown in figures 2 (water) and 6 (sediment).

#### Hydrocarbon analysis

The cleaned hydrocarbon extracts were first analyzed on an Agilent 7890A Series GC (Column: Agilent HP-5MS, 30 m×250 µm×0.25 µm) with Agilent 7890A FID to obtain an estimate of the concentrations and the necessity for dilution. The oven program started at 120°C for 1 min and then was increased to 300°C at 10°C min^−1^, where it was held for 7 minutes. In order to determine the presence and concentrations of PAHs, samples were injected into an Agilent 7890A Series GC (Column: Agilent DB-EUPAH, 20 m×180 µm×0.14 µm) coupled to an Agilent 7000 triple quadrupole MS system with electron ionization source according to a PAH specified multiple reaction monitoring method. The oven program started at 50°C for 0.8 min, then ramped to 180°C at 40°C min^−1^, to 230°C at 7°C min^−1^ and held for 0.5 min, to 280°C at 35°C min^−1^ and held for 0.5 min, and finally to 335°C at 25°C min^−1^ and maintained for 5 min. Helium quenching gas flow was adjusted to 2.25 mL min^−1^, and the nitrogen collision gas flow to 1.5 mL min^−1^. The inlet was kept at 320°C and 27.985 psi. Initial conditions in the column were 50°C, and 27.985 psi, 1.2197 mL min^−1^, 39.998 cm s^−1^ gas flow. The program was concluded with 4 min post run reverse column flushing at −1.48 mL min^−1^. We used a calibration standard with 16 common PAHs (ULTRA SCIENTIFIC PM-610 Polynuclear Aromatic Hydrocarbons Mixture, containing Acenaphthene, Acenaphthylene, Anthracene, Benz[*a*]anthracene, Benzo[*b*]fluoranthene, Benzo[*k*]fluoranthene, Benzo[*ghi*]perylene, Benzo[*a*]pyrene, Chrysene, Dibenz[*a*,*h*]anthracene, Fluoranthene, Fluorene, Indeno[1,2,3-*cd*]pyrene, Naphthalene, Phenanthrene, and Pyrene). Not all of these PAHs were clearly identifiable in our experimental samples, and we report the results of the 12 PAHs that that could be positively identified and were present in significant amounts in our samples.

### In-situ Chamber Measurements

In order to link the findings from the laboratory columns to the natural environment, two in-situ chamber experiments were conducted to quantify hydrocarbon transport into permeable sublittoral sediment. Six advection chambers, with an inner diameter of 19 cm and enclosing a water column of approximately 15 cm height above a sediment surface of 283 cm^2^, were deployed at 1.5 m water depth on sublittoral sands at Pensacola Beach/Florida. The chambers were made of clear acrylic and were stirred by a rotating disc adjusted to produce a radial pressure gradient (0.1 Pa cm^−1^) mimicking the pressure gradients caused by moderate bottom flows (10 cm s^−1^ at 10 cm above the sediment surface) deflected by sand ripples (3 cm amplitude, 30 cm wavelength). In permeable sands, these pressure gradients drive water into and out of the surface layer of the sediment [74]. Further details regarding the advection chamber, its use for measurements in permeable beds, and pore-water flows in permeable sediment can be found in [21], [75]–[77].

In the first chamber experiment, 100 g tarball material was added to two of the six chambers enclosing sand sediment prior to start of the experiment. The tarballs were collected at the study site and consisted of sand grains covered with weathered crude oil from the Deepwater Horizon accident. The oiled sand grains formed small clusters of approximately 5 mm diameter that were distributed evenly over the sediment surface area in the chambers. Two control chambers enclosed sediment without tarball addition. The remaining 2 chambers contained water but no sediment, and to one of these chambers, 100 g tarball material was added. These chambers were used to assess the release of aromatic hydrocarbons from the tarballs under field conditions. During the deployment that lasted 20 h, a Turner Cyclops sensor measured the fluorescence of dissolved organic matter in the water of each chamber. Crude oils contain a wide variety of aromatic species, and these compounds are highly fluorescent, permitting detection of small dissolved or dispersed amounts of aromatic compounds with fluorescence sensors [78], [79]. The fluorescence of aromatic hydrocarbons is reported as equivalents of quinine sulfate, a standard calibration substance for fluorescence measurements containing aromatic quinolone [80]. At the conclusion of the experiment, all water and sediment contained in the chambers was collected for water and sediment analyses.

In the second chamber experiment, three chambers enclosed sediment and water, while the other three chambers contained only water. In each set of three chambers, one chamber was the un-amended control, one received an emulsion of 5 mL M252 oil dispersed in 100 mL artificial seawater (S = 33, created with Instant Ocean®) and one received an emulsion of 5 mL oil dispersed in in 100 mL artificial seawater containing 50 µL Corexit. In the chambers we monitored dissolved organic carbon (DOC) concentrations as proxy for dissolved and colloidal oil particles. Water samples were collected at sunset (19∶30) and sunrise (9∶00) of the following day. At the end of the experiment, all water and sediment contained in the chambers was collected. Analysis of the DOC samples was done on a Shimadzu TOC-V cph machine.

### Statistical Testing

For testing whether differences between our treatments were statistically significant, we used the Mann-Whitney test; and where probability values (p) are reported in the text, this test was applied. The Mann-Whitney test is the non-parametric equivalent of the independent samples t-test, normal distribution of data is not necessary, and it can be used for small samples [81].

## Results

### PAH Release in the Presence and Absence of Corexit

In the absence of Corexit, the PAHs acenaphthylene, anthracene, fluorene and pyrene were released from oil in seawater in quantities above 1 ng ml^−1^ oil within 24 h (Fig. 2). Benzo(g,h,i)perylene, dibenz(a,h)anthracene and indeno(1,2,3-c,d)pyrene were released in smallest amounts, below 0.1 ng ml^−1^ oil within 24 h. PAHs released from the oil increased by roughly a factor 10 for all compounds when Corexit was added, except phenanthrene. For this latter compound the release declined in the presence of Corexit. The difference between PAH release from oil and PAH release from oil in presence of Corexit was statistically significant (p<0.005).

### Short-sand Column Experiment

**Figure 3:**
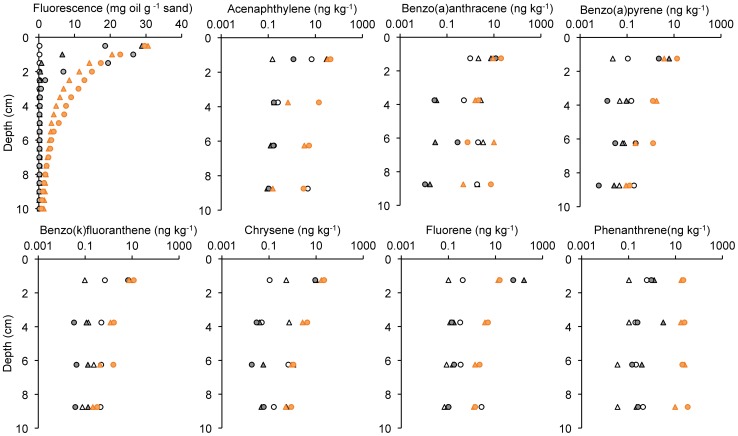
Distribution of fluorescence and depth distribution of PAHs after the Short-Column Experiment. Upper left pane: Fluorescence signal vs. sand depth.; Other panes: PAH concentrations vs. sand depth. The white symbols represent the control experiments, the grey symbols the experiments with oil (treatment I), and the orange symbols the experiments with oil and dispersant (treatment II). Note logarithmic concentration scale in PAH plots, not all PAH species are shown.

The percolation of the 100 ml water through the 10 cm sand columns took approximately 2 minutes, corresponding to a fluid-front velocity of 5 cm min^−1^. This is a velocity that occurs under natural environmental conditions at the beach face [82]. At the concentrations used, the addition of oil or oil and dispersant had no significant effect on the water-percolation rate. After the percolation, the sand columns with crude oil addition but without Corexit showed a sediment surface layer with visible brown oil staining that in the two columns did not exceed a sediment depth of 3 cm. This visual observation was confirmed by the fluorescence measurements that showed high values corresponding to up to 30 mg oil g^−1^ sand in the surface layer decreasing with sediment depth and reaching background values (0.2 mg oil g^−1^) at depth >2.5 cm (Fig. 3). The background fluorescence of uncontaminated sand, as also used in the control columns, corresponded to the fluorescence of 0.2 to 0.3 mg oil g^−1^ sand. Addition of Corexit to the sand at the same concentrations as used for the experiments but without oil, generated fluorescence as would be produced by 0.5 to 0.6 mg oil g^−1^ sand. In the oil-contaminated seawater, flushed through the columns, the addition of the dispersant Corexit increased the dissolved/colloidal PAH concentrations (Fig. 2). In the experiments with both oil and Corexit, the fluorescence signal decreased at a much smaller rate with increasing sediment depth and showed increased values over the 10 cm depth of the sediment, revealing transport of aromatic hydrocarbons through the entire length of the core (corresponding to approximately 1 mg oil g^−1^ sand at 10 cm depth). The measurements of the cores with oil and Corexit were more similar than those of the duplicates of the oil-only experiments, reflecting a more even distribution of the hydrocarbons across the cross section of the cores.

Patterns of the PAH concentrations in the sediment measured using GC/MS supported the patterns detected by the fluorescence method (Fig. 3), but indicated that the different PAHs were transported at different rates through the pore space when dispersants were present. The control sands were characterized by very low oil and PAH concentrations throughout the columns. Where oil was added without Corexit, PAH concentrations in the uppermost sediment layer (0–2.5 cm) were high, while below that layer concentrations were in the same order of magnitude as in the control sands. The PAH distribution in the cores changed when dispersant was present, allowing deeper penetration of these hydrocarbons into the sand as observed for the bulk hydrocarbons. The enhanced transport rate, however, did not affect all PAHs equally: chrysene did not show enhanced concentrations deeper than 4 cm. Acenaphthylene, and benzo(a)pyrene and benzo(k)fluoranthene and fluorene were detected at increased concentrations down to 6 cm, and concentration enhancements in the deepest core section (7.5–10 cm) were found for benzo(a)anthracene and phenanthrene. While trends in the duplicates agreed, the absolute values of the concentrations measured in the depth layers differed.

**Figure 4:**
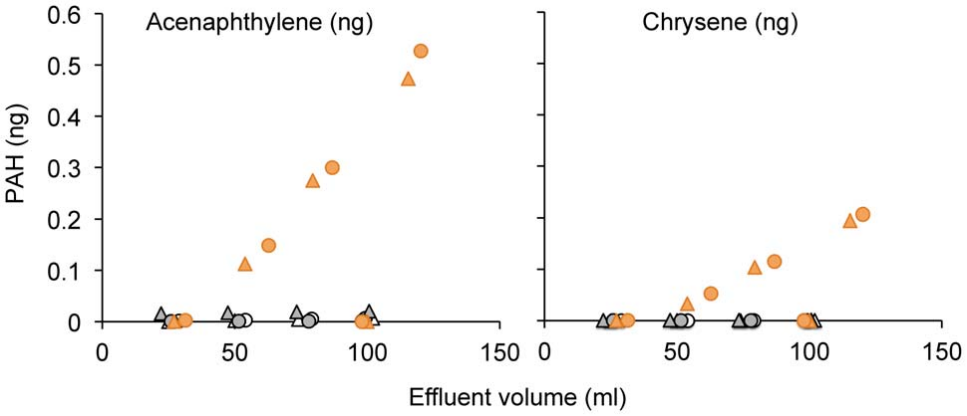
Cumulative release of acenaphthylene and chrysene as a function of the volume of water released from the columns. Triangles indicate the first experiment, circles the duplicate. The white symbols represent the control experiments, the grey symbols the experiments with oil (treatment I), and the orange symbols the experiments with oil and dispersant (treatment II).

The water samples collected at the column outflows supported the results of the core section analyses. The cumulative outflow of PAHs showed that concentrations from the control experiments and the experiments with oil addition but no Corexit remained indistinguishable and close to zero (p>0.005). In contrast, the outflow of the columns with oil and Corexit addition revealed a steady release of PAHs after the initially clean pore water was flushed from the columns with e.g. outflow acenaphthylene concentrations of approximately 6 ng L^−1^ and chrysene concentrations of 2.5 ng L^−1^. The differences between the control columns and columns flushed with water containing oil and Corexit was significant in both runs (p<0.005), and likewise the differences between the columns that received oil and those that received oil and Corexit were highly significant (p<0.005). These results also demonstrated that the relatively low acenaphthylene and chrysene concentrations measured in the deeper sediment sections found don’t allow the conclusion that no chrysene was released from the sediment core. Similar to these two PAHs, GC/MS analysis of the fluid collected at the outflow of the columns treated with oil and Corexit revealed that the PAHs acenaphthylene, anthracene, phenanthrene, pyrene, benzo(a)pyrene, benzo(a)anthracene, dibenz(a,h)anthracene, benzo(ghi)perylene, indeno(123-cd)pyrene, benzo(b)fluoranthene, and benzo(k)fluoranthene, could penetrate through the 10 cm sand columns. Figure 4 shows the cumulative release plots for acenaphthylene and chrysene as examples demonstrating the effect of Corexit on the PAH release from the columns; plots for the other PAHs looked similar. Average concentrations in the 100 mL outflow were below 10 ng L^−1^. Only anthracene had higher concentrations, up to 115 ng L^−1^.

### Long-column Experiment

**Figure 5:**
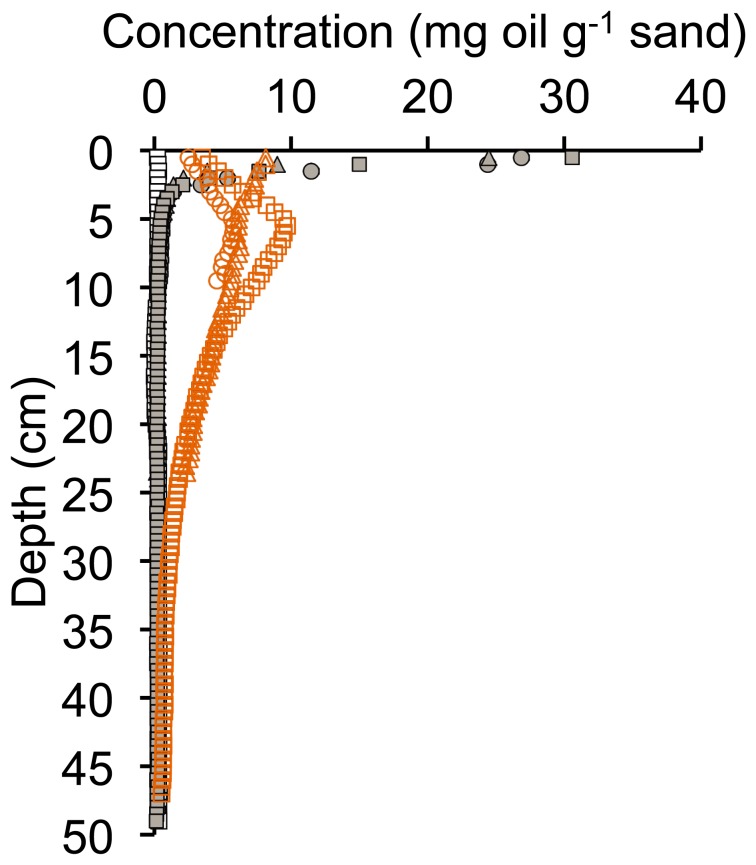
Fluorescence signal of the Long-Column Experiments. The white symbols represent the control experiments, the grey symbols the experiments with oil (treatment I), and the orange symbols the experiments with oil and dispersant (treatment II).

The Long-Column Experiment fluorescence data showed that the increased path length and additional flushing had no measurable effect on the profiles observed for the control experiments and the experiments with oil only (treatment I, Fig. 5). The cores that received oil and Corexit (treatment II) showed oil content above the background value throughout the length of the columns (50 cm). The surface peak that was observed in the Short-Column Experiments, became a subsurface peak in the Long-Column Experiment, with maxima around 5 cm due to the subsequent flushing of clean water after the percolation with the fluids containing oil and Corexit. This finding revealed that the mobilizing effect of Corexit persists even when the flushing water does not contain the dispersant. In the experiments with treatment II, the maximum oil concentration recorded in the sediment layers decreased between the Short- and Long-Column Experiments.

**Figure 6:**
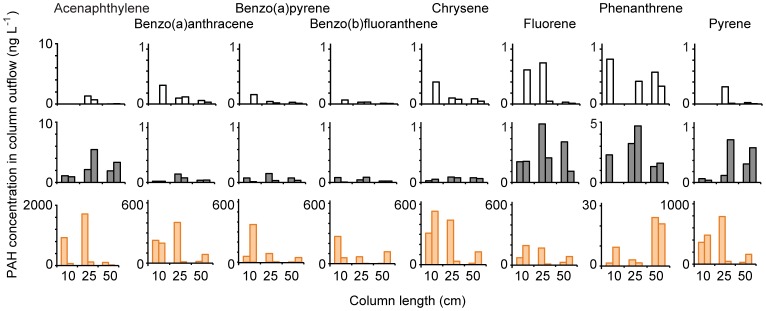
Results of the Long-Column Experiment. Concentrations of different PAHs in the outflowing water from of sand columns with different lengths: upper row) control columns, middle row) columns that received seawater with oil and lower row) columns that received seawater with oil and Corexit. Note the differences in y-axis scaling.

The path length through the sand affected the release of the PAHs in sediment cores with oil and Corexit addition. As found for the short columns, the amount of PAHs measured in the outflow of the long columns treated with oil but without Corexit addition did not differ significantly from the background values found in the outflow of the control columns (Fig. 6, p>0.005). Acenaphthylene was an exception that showed higher concentrations in the column outflows that received oil, however, this difference was statistically not significant. In the treatments with oil and Corexit, all studied PAHs could penetrate through the entire 50 cm of the sand columns, and the PAH concentrations at the outflow were significantly higher than those of the columns with oil but no Corexit (p<0.005), except for Phenanthrene. In general, PAH concentrations in the outflow decreased with column length, however, outflow concentrations showed large variations between parallel columns, in some cases an order of magnitude, obscuring this trend. Unexpectedly, PAH concentrations in the outflow of this Long-Column Experiment were higher than those in the water released from the Short-Column Experiment, which were below 10 ng L^−1^ in all experiments.

On these longer cores we also determined dissolved inorganic carbon (DIC) concentration in the column outflows as an indicator of degradation activity in the columns. The measurements revealed that the DIC concentrations did not differ significantly between treatments and columns, indicating that microbial decomposition activities had no significant influence on the hydrocarbon concentrations that were measured within the columns and the water released from the columns.

### Chamber Experiments

**Figure 7:**
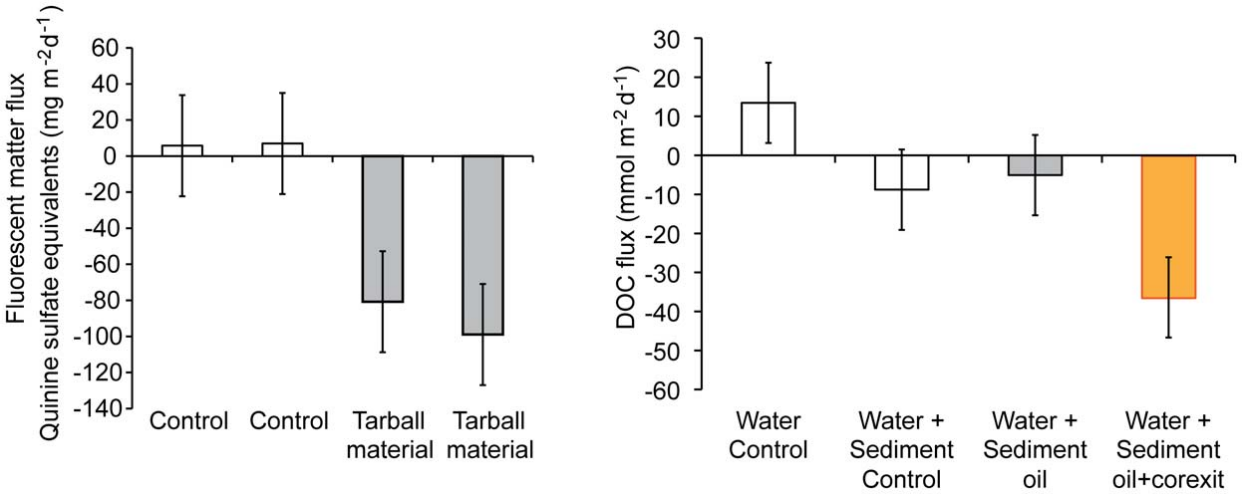
Results from the in-situ chamber incubations. Left pane: chamber experiment 1. No dispersant was applied. Right pane: Results from chamber experiment 2. The DOC measurements were less sensitive than the fluorescence measurements and included all dissolved organic carbon. Error bars depict standard error.

In the first experiment, the tarball material in the chamber containing water but no sediment caused a fluorescence increase in the water of 17.5 µg L^−1^ h^−1^ (Quinine sulfate equivalents), while in the chamber without tarball material, natural chromophoric dissolved organic matter (CDOM) generated by bacteria or algae produced an increase of 0.6 µg L^−1^ h^−1^. Subtraction of these water fluorescence data from the fluorescence data recorded in the chambers containing sediment and water revealed a sedimentary release of fluorescent substances (5.8, 7.0 mg m^−2^ d^−1^ (Quinine sulfate equivalents)) in the control chambers and an uptake of fluorescent substances (−80.8, −99.0 mg m^−2^ d^−1^) by the sand in the chambers with tarball material (Fig. 7a). We concluded from these results that aromatic compounds released from the tarball material scattered on the surface of the enclosed sediment were transported into and absorbed by the surface layer of the sand.

In the second experiment, the stirring motors of two chambers of the set with water but no sediment failed so that only results for the control chamber without additions could be obtained. In the chambers with water and sediment, the measurements revealed that DOC fluxes were directed into the sediment, and the fluxes in the chamber containing oil and Corexit exceeded those in the control chamber four times. The DOC flux in the chamber with oil but no Corexit did not differ significantly from the DOC flux in the control chamber (Fig. 7b). We concluded from this experiment that the addition of Corexit increased the flux of dissolved or colloidal oil components into the sediment.

## Discussion

Our column experiments show that the addition of dispersant increases the mobility of PAH in permeable saturated Gulf of Mexico sands, and the chamber experiments indicate that aromatic oil components can be transported from the water column into natural sand beds. Within the sediment, the transport rate of PAHs exceeded that of the total petroleum hydrocarbon peak, which is attributed to increased PAH partitioning into water- and colloid-facilitated transport [37]. We found that the application of the dispersant Corexit increased the dissolved/colloidal PAH concentrations in MC252-oil-contaminated water, which supported the enhanced mobility of the PAHs through the sand (Fig. 2). An increase of dissolved PAH concentrations in contaminated water after dispersant application was also observed in mesocosm experiments conducted by Yamada et al. [26].

The highest PAH concentrations in the column experiments with only oil were at most 70% of the highest concentration observed in the presence of Corexit. Exceptions to this pattern were found in the short columns for fluorene and anthracene, where the highest concentrations were recorded in the experiments with only oil. It could be expected that the PAH concentrations would be highest in the upper sediment layer of the sediments that were flushed by water containing oil but not dispersant, considering that the oil in these columns was retained in the upper 2.5 cm. The observation that the PAH concentrations, except the mentioned exceptions, in these columns were always lower than in the columns that received oil and Corexit, emphasizes the effect of the dispersant in mobilizing PAH through transferring them to dissolved and colloidal fractions. The PAH concentrations in the replicate cores with oil and Corexit were more similar than those of the duplicates of the oil-only experiments, reflecting a more even distribution of the hydrocarbons across the cross section of the cores. This may be related to the smaller and thus more mobile particles formed when dispersant is present.

**Figure 8:**
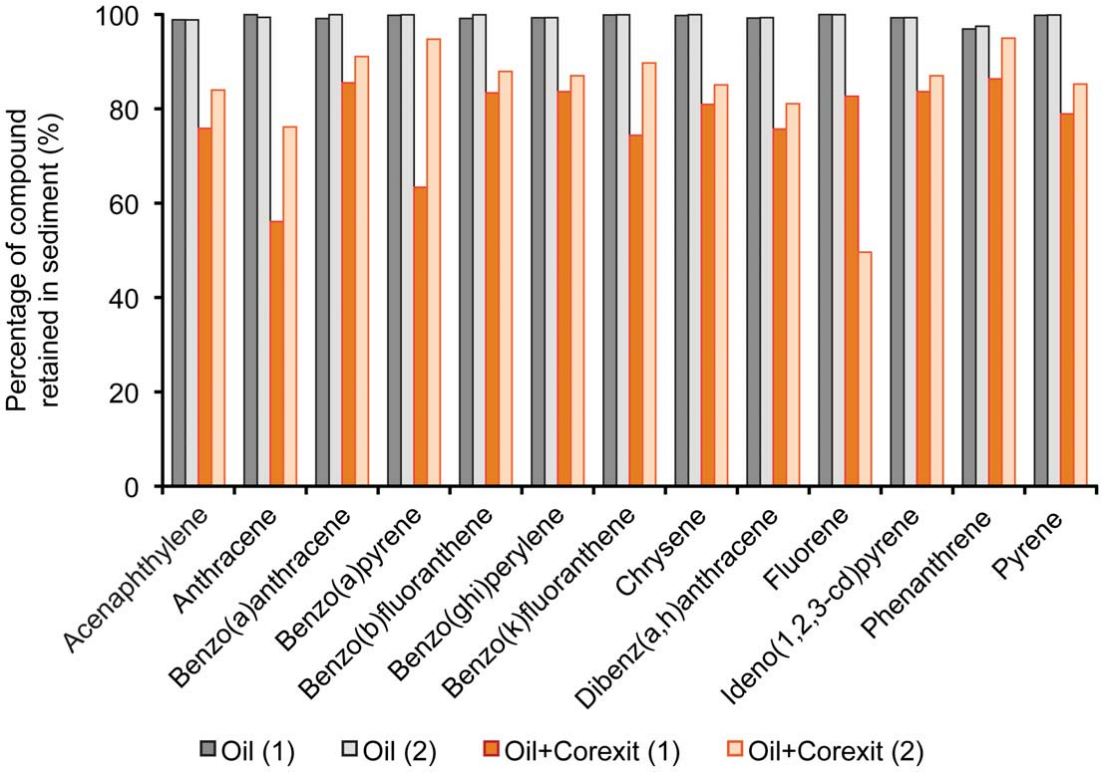
PAHs retained in the sand during the Short-Column Experiment. The columns show the percentage of PAH that was retained in the sediment of the total PAH amount added to the Short-Column Experiments.

Using mass balance calculations on the data of the Short-Column Experiments, we found that over 97% of the target PAHs were retained in the sediment when no dispersant was added to the oil. This percentage dropped on average to 80% in the presence of Corexit, and the difference between treatments I and II was significant (P<0.005). Fluorene retention decreased to 50% (Fig. 8). It should be noted that these values reflect retention after a single flushing event.

The causes of the reduced PAH retention after dispersant application has several reasons: 1) the dispersant transforms the oil containing the PAHs into small micelles that can penetrate through the interstitial space of the sand [83], [84]. 2) the coating of the oil particles produced by the dispersant reduces the sorption to the sand grains [85], 3) saline conditions enhance the adsorption of dispersant to sand surfaces, thereby reducing the sorption of oil to the grains [86].

**Figure 9:**
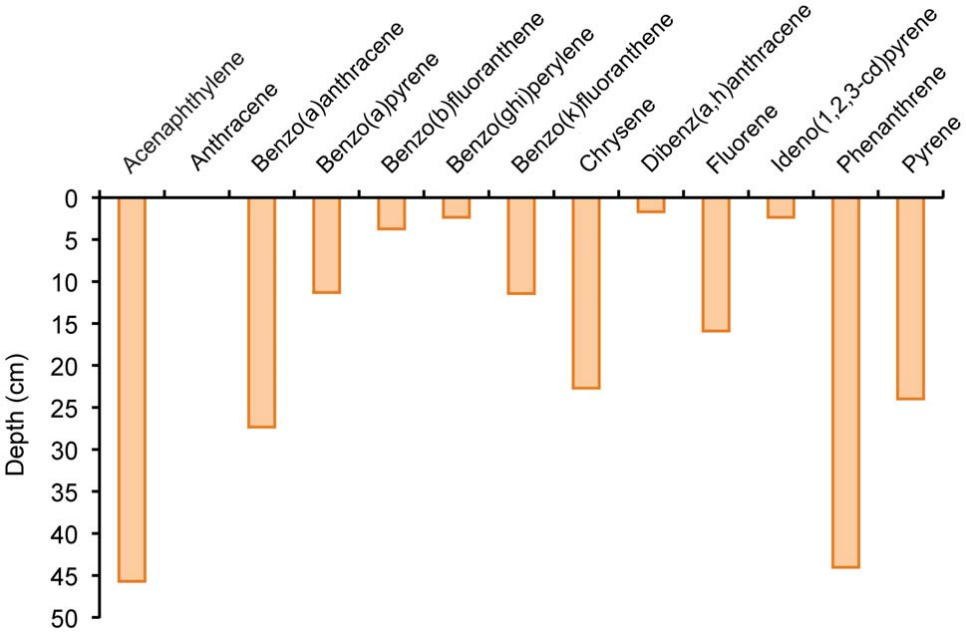
Calculated penetration depths for oil concentrations and PAH measured in our Short-Column Experiment one in the presence of Corexit. Depths were extrapolated from the of PAH concentrations in the sediment sections assuming an exponential trend. Anthracene data did not allow calculation of a trend due to large scatter of data.

From the PAH depth distributions recorded in the Short-Column Experiment as shown in Figure 3, it is possible to infer a hypothetical penetration depth by plotting a trend line through the concentration vs. depth data, and assuming that the concentration decrease with depth follows an exponentially decreasing trend (Fig. 9). The penetration depth was defined as the depth where the extrapolated trend line reached the concentration 0.1 ng kg^−1^.

**Figure 10:**
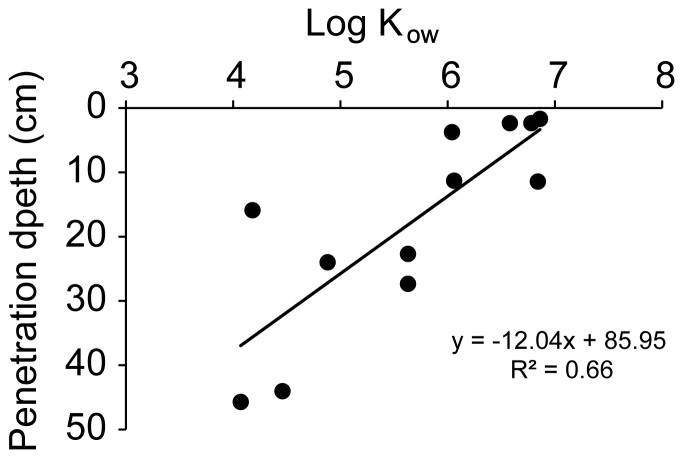
Relationship between inferred penetration depth and the hydrophobicity (log K_ow_) of the PAHs. K_ow_ = Concentration in octanol phase/Concentration in aqueous phase. The K_ow_ values for the PAHs were retrieved from http://www.env.gov.bc.ca/wat/wq/BCguidelines/pahs/pahs-01.htm.

These estimates reveal differences in the penetration behavior of the different PAHs and show the depth range that can be reached by these substances after a single flushing event, e.g. as caused by a wave washing onto an oil contaminated beach face. A main factor that defines the penetration of specific PAHs through saturated sand is the hydrophobicity of the molecules [87], i.e. the tendency of the hydrocarbons to form intermolecular aggregates in the aqueous medium and to adsorb to non-polar surfaces [88]. As a measure for hydrophobicity, we used the octanol-water partition coefficient (K_OW_), which represents the ratio of the solubility of the hydrocarbon in the non-polar solvent octanol to its solubility in the polar solvent water [89]. The Log K_OW_ values, which are inversely related to aqueous solubility and directly proportional to molecular weight, are a relative indicator of the tendency of an organic compound to adsorb to sediment [85]. The lower the Log K_OW_ values for the PAH, the greater was its penetration depth in the saturated sand. The PAH penetration depths calculated for our column experiment were negatively related to the hydrophobicity (r^2^ = 0.66), showing that more hydrophobic compounds are better retained in the sands (Fig. 10). The least hydrophobic PAHs (acenaphthylene, phenanthrene) penetrated deepest into the sand.

However, in the Long-Column Experiment, detectable PAH concentrations were recorded in the outflow of all columns, and estimates of penetration depths, here calculated through extrapolation of the outflow concentrations of the columns with different lengths, revealed that all PAHs could penetrate roughly 50 to 80 centimeters into the sand. These findings disagree with the results of the Short-Column Experiment, where the mass balance results show that 80% of the oil is retained in the upper 10 cm, and calculated penetration depths of PAHs were limited to 50 cm into the sand. This apparent disagreement can be explained by the additional flushing applied in the Long-Column Experiment after the initial percolation with oil and Corexit contaminated water. Oil can move more easily through sand at low aqueous oil concentrations [90] and, thus can penetrate deeper into the sediment with repeated flushing events. In the Long-Column Experiment, some of the oil that was retained in the uppermost sediment layer after the passage of oiled water was remobilized by the second water surge, moving the sedimentary hydrocarbon concentration peak deeper within the sand despite the fact that the clean seawater did not contain oil or dispersant. Saline conditions, as in our experiments, support the adsorption of dispersant to the surface of the sand grains [86], producing a lining of the pore space that facilitates deeper penetration of PAHs during consecutive flushing events even with uncontaminated sea water. This finding suggests, that repeated flushing as caused by waves washing up a contaminated beach may pump PAHs deep into the sediment when dispersant is present. Natural dispersants, as produced by oil-degrading bacteria [91], may support this effect when oil is present in the sand for longer time periods. This process also may partially explain the deep penetration of PAHs reported by Bernabeu et al. [92] from the Spanish coasts, where increased PAH concentrations were recorded at 1 m below the oil concentration peak in sandy beach sediment.

The continuous flushing by waves washing up an oil-contaminated beach may result in the release of PAH from the beach sediment back to the coastal water with the pore water flows draining the beach [93], [94]. After PAHs are released from the sediment, exposure to UV-light can increase PAH degradation [95] but also toxicity to marine biota five- to eightfold [96], [97]. Calculation of potential PAH release from a 1 m wide beach face area, based on the results of the Short-Column Experiment and assuming a water release rate of 3000 L m^−1^ h^−1^; [98], suggest that the PAH release can increase substantially when Corexit is present (Table 2).

**Table 2:** Effect of dispersant of the potential release rates of PAHs from a sandy beach (1 m wide beach face area) based on the results obtained in the Short-column experiment.

	No oil or Corexit		1 L oil per m^2^		1 L oil+Corexit per m^2^	
	Run 1	Run 2	Run 1	Run 2	Run 1	Run 2
PAH	mg m^−1^ d^−1^		mg m^−1^ d^−1^		mg m^−1^ d^−1^	
Acenaphthylene	0.5	0.5	1.4	0.0	34.1	37.9
Anthracene	0.5	0.7	1.0	0.2	384.0	221.5
Benzo(a)anthracene	0.0	0.0	0.2	0.0	11.3	8.9
Benzo(a)pyrene	0.0	0.0	0.0	0.0	10.1	2.9
Benzo(b)fluoranthene	0.0	0.0	0.0	0.0	1.9	2.2
Benzo(k)fluoranthene	0.0	0.0	0.0	0.0	9.1	5.5
Chrysene	0.0	0.0	0.0	0.0	13.9	14.9
Dibenz(a,h)anthracene	0.0	0.0	0.2	0.0	2.2	1.7
Fluorene	0.2	0.2	0.2	0.0	11.8	71.3
Indeno(123-cd)pyrene	0.0	0.0	0.2	0.0	1.4	1.4
Phenanthrene	0.2	0.2	0.5	0.2	34.1	15.4
Pyrene	0.5	0.5	0.0	0.0	17.8	17.0
Total PAH release	1.9	2.2	3.8	0.5	531.6	400.6

The oil concentrations used in our experiments are at the lower end of those reported for coastal waters after the Deepwater Horizon accident [20], and the Gulf of Mexico beaches were flooded with consecutive surges of oil, which inevitably lead to higher oil concentrations in the surface sediments [99]. Ensuing oil layers can persist in coarse beach sediments for decades [100], and flushing by waves may leach PAHs from these layers. Our column experiments demonstrate that the application of dispersants, as performed extensively after the Deepwater Horizon oil spill, can significantly enhance the release of PAHs from embedded oil layers and facilitate the transport of the PAHs into deep sediment layers. The beaches, however, may not be the only sites where the transport processes described here are relevant. The continuous washing up of tarballs onto Florida Beaches suggests that larger buried oil layers still may exist in the shallow shelf of the northeastern Gulf of Mexico [20]. This area is covered by relatively coarse sands that are flushed by bottom currents that are deflected by the ripple topography of the sea bed [74], [101]. The advection chambers we deployed in this area simulate the sedimentary flushing by the currents and waves. The in-situ incubations showed that tarballs on the sediment surface can release aromatic hydrocarbons that are transported into the highly permeable sediment. The flux of hydrocarbons into the sediment was higher in the presence of dispersant. These results suggest that the findings from the column experiments are also applicable to sublittoral settings, where PAHs released from buried oil layers may be pumped deeper into the sediment with current-driven pore water flows. Exclusion from oxygen in the deeper sediment layers can significantly reduce the degradation rate of hydrocarbons [102] thereby extending the persistence of the PAHs in the marine environment.

### Conclusions

Our experiments suggest that as a consequence of addition of Corexit 9500A to spilled MC252 oil after the Deepwater Horizon accident, PAHs from this oil source were able to penetrate deeper into the sandy beach sediments of the Northeastern Gulf of Mexico than would have been possible in the absence of dispersant. Our in situ chamber deployments indicate that dispersants also can increase the sediment-water-flux of PAHs in sublittoral environments. Where dispersants facilitate a deeper penetration of hydrocarbons into marine sediments, these hydrocarbons including PAHs may end up in anoxic sediment layers due to the natural spatial and temporal variability of advective pore water flows. This lack of oxygen may slow the PAH degradation rates, enhancing the environmental lifetime of these potentially harmful compounds. On the other hand, the dispersion of the hydrocarbons in oxygenated sediment layers facilitated by Corexit 9500A may also make them available to a larger number of microbes and thereby may enhance biodegradation and pollutant mass removal.
